# Supplementary material for: Learning curve and surgical outcome of robotic assisted colorectal surgery with ERAS program
Source: Sci Rep. 2022 Nov 29;12:20566. doi: 10.1038/s41598-022-24665-w (PMC9709162; doi:10.1038/s41598-022-24665-w)
Supplement: Supplementary file 2 — Supplementary Information 2. [file 41598_2022_24665_MOESM2_ESM.zip › RAL Raw data-Ñ[▒K/055/OP_ñw╝╨░O▒Kñσ.pdf]

正式摘要(電子簽章：000000208879602)

W95 / W95

臺中榮民總醫院

Operation Note

[redacted]

- 手術日期：2021/01/23
- 手術醫師：VS 蔣鋒帆，CR 陳映菁，R 李一宏
- 執行麻醉：VS 蔡志忠
- Anesthesia : GE
- Position : Lithotomy
- Aseptic agents : Soap , Betadine Alcohol , Betadine Aqua

Diagnosis

- Pre-operative diagnosis : Malignant tumor of rectum, s/p CCRT
- Post-operative diagnosis : Ditto.

Surgical Indication

- curative treatment

Operation

- 1. Robotic (XI) assisted TME+ loop ileostomy
  2. Intraoperative colonoscopy : (-).
  3. High ligation (+) : At IMA root and IMV (Via robotic hemolok).
  4. Take down from the splenic flexure : (-)
  5. Robotic EndoGIA (45-4.3) X2 + EEA (29mm) + OpenGIA (80-3.8) X1 were applied to complete the side-to-end anastomosis
  6. Proximal cut end: 19 cm, distal cut end: 1.5 cm
  7. No drainage tube was inserted
  8. ADHESION BARRIER FILM was placed
  9. Loop ileostomy (About 15cm from IC valve) was done over RLQ of abdomen

Finding

- 1. A 4 x 5 cm tumor at lower rectum. (DRE : Finger tip could reach the lower edge of the tumor)
  2. No other gross tumor nor polyps was found
  3. There was no obvious visible liver metastasis, no visible peritoneal seeding
  4. No obvious ascites was noted
  5. Bilateral doughnuts : intact ; Air leak test: Done and no air leak was found

Procedure

- 1. General endotracheal anesthesia was induced and then the patient was placed in modified Trendelenburg lithotomy position.
  2. Preoperative antibiotics were given. A Foley catheter and nasogastric tube were placed.
  3. The abdomen was prepped and draped in standard sterile fashion.
  4. A small vertical incision was made above the umbilicus and a 8 mm trocar was inserted into the peritoneal cavity by open method. Peumoperitoneum was established with CO2 inflation.
  5. Laparoscopy was inserted and then other two 8mm trocars were inserted over LUQ, RLQ of abdomen. Another 12mm trocars was inserted over RLQ area, and the other assist-port 5mm troca was inserted on RUQ. The operative findings were mentioned as above.
  6. Lateral to medial approach was done due to moderate adhesion. White line of Toldt was recongnized and dissection was done above Georta fascia and toward the peritoneal attachments
  7. The sigmoid colon was held by grasper and the peritoneum overlying the mesentery was scored with electrocautery.

- 8. The inferior mesentery artery was identified, ligated above the left colic artery with robotic clips, and transected. IMV was ligated via hemolok and then also transected.
  9. The colon was mobilized from retroperitoneum overlying the sacral promontory and into the pelvis.

  10. Distal descending colon and s-colon were freely mobilized after the mesoclon was all dissected.
  11. The incision was then extended laterally and medially across the peritoneal reflection.
  12. The mesorectum was then mobilized using sharp dissection over the level of pelvic floor muscle. Then rectum was mobilized as possible.
  13. DRE was performed at the same time to make sure the desired resection line is adequate.
  14. The distal rectum was divided with robotic endoGIA (45-4.3) X2.
  15. The umbilicum area wound of abdomen was further extended. We approached into the peritoneal cavity by open method. S-colon and rectum was pulled out.
  16. The distal s-colon was then taken out for carefully inspection to make sure it has an excellent blood supply. The mesenteric fat was gently cleared from the terminal 2-3 mm of the bowel ends.
  17. The field was isolated with moist pad and the bowel opened and sized. It was determined that a 29 mm circular stapler could be accommodated. A small incision was made near the end of the s-colon, which then gentally extended to allow the anvil to be inserted. The anvil was penatrated from the lateral side of the s-colon. (For later side to end approach)
  18. Then the anvil was fixed with mersilk sutures. The residule terminal end of the s-colon was sealed and resected via firing OpenGIA (80-3.8) X1
  19. The colon was then put back to the abdominal cavity and pneumoperitoneum was constructed again.
  20. The anal canal was irrigated with distil water and then the circular stapler was introduced through the anus.
  21. The proximal bowel with anvil in place (Side) was engaged with the circular stapler (End) and the stapler was then fired. Two complete donuts were obtained and the distal one was submitted to pathology for margin confirmation.
  22. The anastomosis was tested for leaks with irrigation solution and air introduced through the rectum and was found to be patent and intact.
  23. The bleeder was checked thorughly again.
  24. The desired ileum site (About 15 from IC valve) was selected for further ostomy construction.
  25. The 5mm assistant trochar wound over the RUQ area was further extended to creat the site for loop ileostomy.
  26. The ileum was retracted from the desired ostomy site. A window was created under the ileum by dividing the mesentery adjacent to the bowel wall with electrical cautery carefully, with a plastic bridge in placed.
  27. The trochar fascia wounds were all then closed with interrupted sutures via 1-O Vicryl.
  28. ADHESION BARRIER FILM was placed below the central abdominal wound.
  29. The extended fascia wound was closed with interrupted sutures via Num.1 Vicryl.
  30. Massive N/S was applied to irrigate the wound.
  31. The wound was further closed with 3-O Vicryl, and the skin was applied with SurgiSeal.
  32. Moist pads were placed and the ileum was opened. Succus and air were aspirated.
  33. The ileostomy was matured with multiple interrupted mucocutaneous suture via 4-0 Monocryl.
  34. The ileostomy was well formed and viable. An ostomy bag was placed
  35. The patient tolerated the procedure well and was brought to the POR in stable condition.
- Estimated blood loss: minimum
- 器械數量: 正確
- 紗布數量: 正確

Image

- PAINT
  - colon & rectum

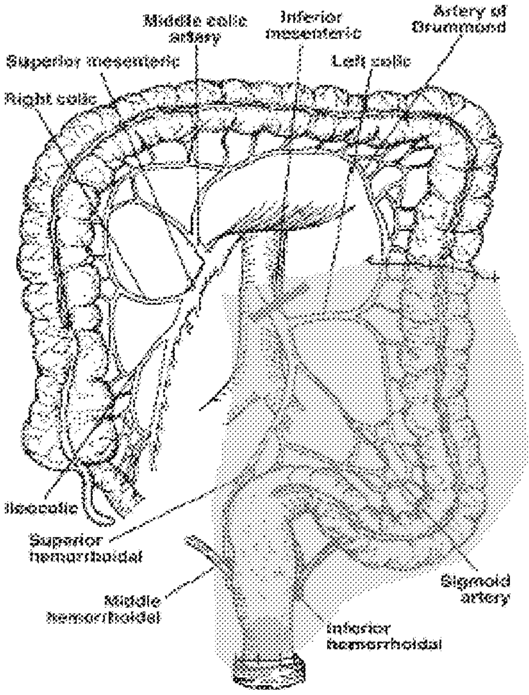

- abdomen

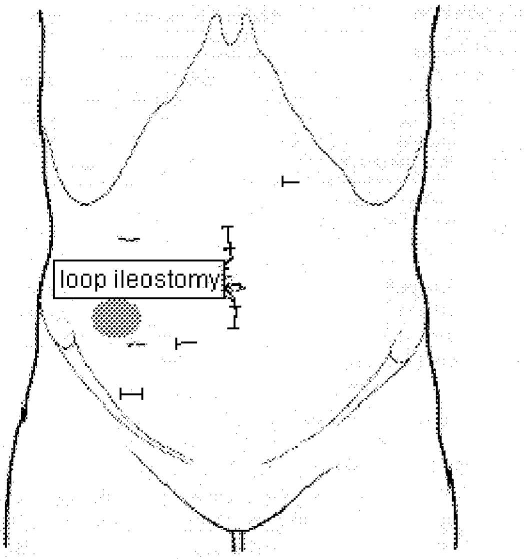

主任 2062F蔣鋒帆 / 醫師 2303K陳映菁

正式摘要**(電子簽章：000000208879602)**

W95 / W95

doctorRank:B
